# Towards a core outcome set (COS) for intrinsic capacity (IC) intervention studies in adults in midlife and beyond: a scoping review to identify frequently used outcomes and measurement tools

**DOI:** 10.1007/s40520-023-02681-8

**Published:** 2024-03-05

**Authors:** Dolores Sanchez-Rodriguez, Olivier Bruyère, Murielle Surquin, Jean-Yves Reginster, Charlotte Beaudart

**Affiliations:** 1 Geriatrics Department, Brugmann University Hospital, Université Libre de Bruxelles, Brussels, Belgium; 2 WHO Collaborating Centre for Public Health Aspects of Musculo-Skeletal Health and Ageing, Division of Public Health, Epidemiology and Health Economics, University of Liège, Liège, Belgium; 3 Geriatrics Department, Rehabilitation Research Group, Hospital Del Mar Research Institute, Barcelona, Spain; 4 Clinical Pharmacology and Toxicology Research Unit, Faculty of Medicine, NAmur Research Institute for LIfe Sciences (NARILIS), University of Namur, Namur, Belgium

**Keywords:** Core outcome set, ICOPE, Intrinsic capacity, Scoping review, Older people

## Abstract

This scoping review was conducted to identify the outcomes and measurement tools used in IC intervention studies, as first step towards the development of a core outcome set (COS) for IC trials. PRISMA-ScR and COS-STAD were followed. The review considered randomized controlled trials targeting IC published in Medline, Scopus, Embase, Cochrane Central Register of Controlled Trials, and clinicaltrials.gov, until June 2023. Of 699 references, 534 studies were screened once duplicates were removed, 15 were assessed for eligibility, and 7 (4 articles and 3 protocols) met eligibility criteria. Twenty-eight outcomes were identified (19 related to IC and its domains and 9 unrelated). The most reported primary outcome was the change in IC levels postintervention (5 over 7 studies) and the most reported outcomes (either as primary and/or secondary) were the changes in physical performance and in depressive symptoms (6 over 7 studies). Fifty-five tools used to construct the domains’ *z*-scores and/or assess the effect of interventions were identified (47 related to IC and its domains and 8 unrelated). The most reported tool was an IC *Z*-score, calculated by 4 domains’ *z*-scores: locomotor, vitality, cognitive, and psychological (5 over 7 studies). The tools differed among studies (10 locomotor related, 6 vitality related, 16 cognitive related, 8 psychological related, 6 sensorial related, 8 unrelated tools). The vast heterogeneity (28 outcomes and 55 tools within 7 studies) highlighted the need of a COS. These outcomes and tools will be presented to experts in a future step, to select the ones that should be taken into consideration in IC trials.

**Supplementary Information:**

The online version contains supplementary material available at 10.1007/s40520-023-02681-8.

## Introduction

Intrinsic capacity (IC) is the composite of all the physical and mental capacities of an individual, an emerging Public Health indicator, developed by the World Health Organization (WHO) in 2016. IC aims to preserve health in mid-life and beyond, towards a better aging process [1]. There is growing interest about IC due to its ability to anticipate health outcomes [2, 3]. The dynamic nature of IC, and the potential reversibility of the losses of IC and its individual domains, point out this health indicator as an adequate target both for person-centered and Public Health interventions [4]. There is growing evidence about the potential benefits that interventions in IC may bring to older populations, e.g., in terms of enhancing the locomotor, vitality, cognitive, and psychological domains [5]. Moreover, in recent times, the potential of IC for fragility fracture prevention has been pointed out [6].

The Core Outcome Sets (COS) are evidence and consensus-based “standardized collections of outcomes to be measured and reported, as a minimum, in the clinical trials about a specific condition or disease” [7]. The development of COS follows standardized procedures and is highly desirable, to identify meaningful outcomes, harmonize their use in clinical trials, facilitate pulling data together in form of meta-analysis and network meta-analysis, and enhance transparency in research [7]. More than 370 COS have been developed in the latest years, and very few of them concern interventions in older people, e.g., the COS for malnutrition [8], sarcopenia [9, 10], and frailty [11] intervention studies in older people have been recently developed [12]. However, to date, as IC is a relatively new construct, no COS for IC intervention studies is yet available. It would be crucial to launch the COS in IC interventions after some evidence is available, and before many IC intervention studies are developed. This is the right time to do that, to ensure that future intervention studies will adhere to this COS, and that the COS meets its purpose and is helpful for the clinical and scientific community interested in IC and Healthy aging.

The standardized procedures for COS development have been created and are among the guidelines of the Enhancing the QUAlity and Transparency Of health Research (EQUATOR) Network (https://www.equator-network.org/), supported by the WHO, among others, which provide minimum standard operational procedures for the design [7], protocol drafting [13], and final reporting [14] of COS. Among the COS-STAD recommendations, one of the initial steps, crucial for the methodological quality of the COS, is conducting a systematic search about the reported outcomes in existing literature [7]. Therefore, there is an urgent need to identify the outcomes investigated in interventions in IC, to develop a COS for IC intervention studies in older people. As one of the first recommended steps towards developing a COS [7], a scoping review (ScR) to provide an overview of outcomes and their measurement tools was conducted. The objective of this review is to systematically identify all the outcomes and their measurement tools investigated in RCT aiming at the management of IC in older people, using any type of intervention.

## Methods

This ScR followed the Preferred Reporting Items for Systematic Reviews and Meta-Analyses extension for Scoping Reviews (PRISMA-ScR) (Completed checklist is available in Supplementary material, Table 1S) [15]. The COS-STAD for the design of a COS study was followed as far as possible [7, 13, 14]. The protocol was registered in the International prospective register of systematic reviews (PROSPERO, CRD42023437223), Open Science Framework, and the Core Outcome Measures in Effectiveness Trials (COMET) database on June 2023.

A working group of collaborators was gathered under the auspices of the WHO Collaborating Centre for Public Health Aspects of Musculo-Skeletal Health and Ageing, University of Liège, Liège (Belgium) and the European Society for Clinical and Economic Aspects of Osteoporosis and Osteoarthritis (ESCEO).

### Population/concept/context (PCC)

The PCC of the ScR are shown in Table 1.

**Table 1:** Inclusion criteria of the scoping review (population/concept/context, PCC)

Population	Target in midlife and beyond, with no limitations related to age Any satisfactory or unsatisfactory status of Intrinsic capacity (IC) (and any satisfactory or unsatisfactory domains) Any healthcare geriatric setting (community-dwelling, nursing home, and hospitalized population) Healthy population or in presence of any condition and/or disease
Concept	Randomized controlled trials (RCT) aiming at the management of IC in older people, using any type of intervention *All-outcomes (primary and secondary outcomes)*
Context	Articles published from inception of the term IC (2016) to June 16, 2023 (date when the last bibliographic search was consulted). No language restriction was applied Full-text, peer-reviewed publications in indexed journals, congress abstracts, and letters to editor containing original data. Grey/unpublished literature, i.e., clinical trial registries (ClinicalTrials.gov), were consulted to verify if some RCT had been conducted with results not reported in literature. Authors were contacted to obtain missing data

*IC* intrinsic capacity, *RCT* randomized controlled trial

Exclusion criteria:

- Animal studies.
- Observational studies, case reports, reviews, and letters to the editors (unless they contained original data).

### Search strategy and study selection

Four bibliographic databases, i.e., Medline (via Ovid), Scopus, Embase, and Cochrane CENTRAL Register of Controlled Trials (via Ovid) databases were searched until June 2023. The search strategies used for each bibliographic databases were developed in collaboration with an experimented librarian researcher and are available in Supplementary material, Table 2S*.*

A manual search within the references of relevant articles was performed to complete the bibliographic search (backward citation searching). Moreover, Web of Science was used to identify any other research that has referenced any of the articles of interest (forward citation searching). As it is a ScR, previous systematic reviews and meta-analyses on a similar topic were also investigated. Clinical trial registries (www.clinicaltrial.gov) were explored for potential unpublished trials. Experts on the field were contacted to provide valuable input on the search strategy, the full-text articles unavailable in any institutional bibliographic sources, and to provide potential missing studies.

The results retrieved from the search within the electronic sources and manual searching were imported to Covidence software for data management. All identified articles were screened for their eligibility by two independent reviewers (DSR & CB) first based on their titles and abstracts and second, based on their full-text articles. Disagreement among the two reviewers was solved by consensus between the two reviewers or by intervention of a third reviewer, if necessary (OB).

### Data extraction

A standardized data extraction form was generated and used for data extraction by the two independent reviewers. The reviewers (DSR & CB) who conducted the systematic search and the article selection process recorded and synthesized from each full-text article the relevant information related to the review. The following data were extracted: information related to the study (author, year of publication, journal), information related to the intervention (groups, type of intervention, length of the intervention, length of the follow-up), information related to the outcomes (primary and/or secondary outcomes), and the measurement tools (clinically meaningful significant change, substantial change, if available, etc.). Outcomes were considered as primary or secondary based on the information in the original texts. The authors of individual articles were contacted in case of missing information. A summary table was drafted, exposing the findings of the search in chronological order (newest studies first) and synthesizing data for each study.

### Risk of bias assessment

The risk of bias assessment of individual studies was not conducted because it is an optional item in PRISMA-ScR [15] and because the ScR was not looking at the results of the RCTs, but only at the outcomes and tools used within those RCTs.

### Strategy for data synthesis

Results were presented using qualitative synthesis. Due to the nature of data investigated, no meta-analysis was undertaken. In trials which explicitly defined outcomes, those outcomes were listed as quoted in the original studies. However, for trials where outcomes were not explicitly mentioned or defined by their measurement tools instead, the tools were rephrased based on the outcomes they represented. This decision was made to ensure consistency in the ScR synthesis process, considering the lack of consensus in the terminology for IC, and the inclusion of protocols. For transparency, the ScR synthesis table includes both the terms used for the outcomes as quoted in the original studies, and the rephrased terms used for ScR purposes, in two separate columns. The outcomes were divided into two groups: outcomes related to IC and its domains and outcomes unrelated to IC and its domains.

The measurement tools were recorded exactly as they appeared in the original texts: i.e., those tools that are formed by the combination of several components (e.g., SPPB), and were mentioned as the total score, were recorded as quoted. Likewise, those trials which took into consideration each separate component of the tool (e.g., 4-m gait speed test, balance test, and Timed Chair Stand test, with/without the total SPPB score) were also recorded exactly as described in the original studies.

The IC *Z*-score mentioned in the ScR and some of the trials refers to a statistical measurement of IC levels. It aims to reflect the IC as a global construct, representing the distance of the studied population from the mean, expressed in standard deviations, and calculated based on the domains’ *z*-scores [3]. The *Z*-score was considered as a tool in this ScR. This decision was made because the outcome of these trials was defined as “the change in global IC levels postintervention’’, and IC was measured by the *Z*-score. Thus, the IC *Z*-score served as the IC measurement tool.

In studies using the IC *Z*-score as the tool, the calculation necessarily involves the domains' *z*-scores as an implicit, mandatory intermediate step. The rationale for including the intermediate step (domains' *z*-scores) among the measurement tools was to provide a detailed account of all elements required to construct the IC *Z*-score. This level of detail was considered relevant for ScR and future COS purposes, especially since the future COS might include multiple tests to assess each domain. The potential combination of these tests in different ways demands that the construction of the domains’ *z*-scores be explicitly listed in the ScR and taken into consideration during COS development.

In summary, the following elements have been considered and listed under the term “tools”:

- IC *Z*-score (calculated based on the domains’ *z*-scores).
- Domains’ *z*-scores (e.g., locomotor *z*-score, vitality *z*-score, etc.).
- The tools to construct the domains’ *z*-scores (these may be equal to the domain *z*-score if only one test is used or different if multiple tools are used within a domain). An explanatory diagram is available in Supplementary material, Fig. 1S.

For ScR purposes, the tools were categorized based on the five IC domains they were related to, with an additional sixth category summarizing those tools unrelated to IC and its domains.

The outcome list (either as primary and/or secondary), and the 6 tool categories were graphically represented by their corresponding clustered bar charts, which showed the frequency values within each category in the RCTs, and were ordered based on their frequency values (most frequent tools first).

## Results

**Fig. 1:**
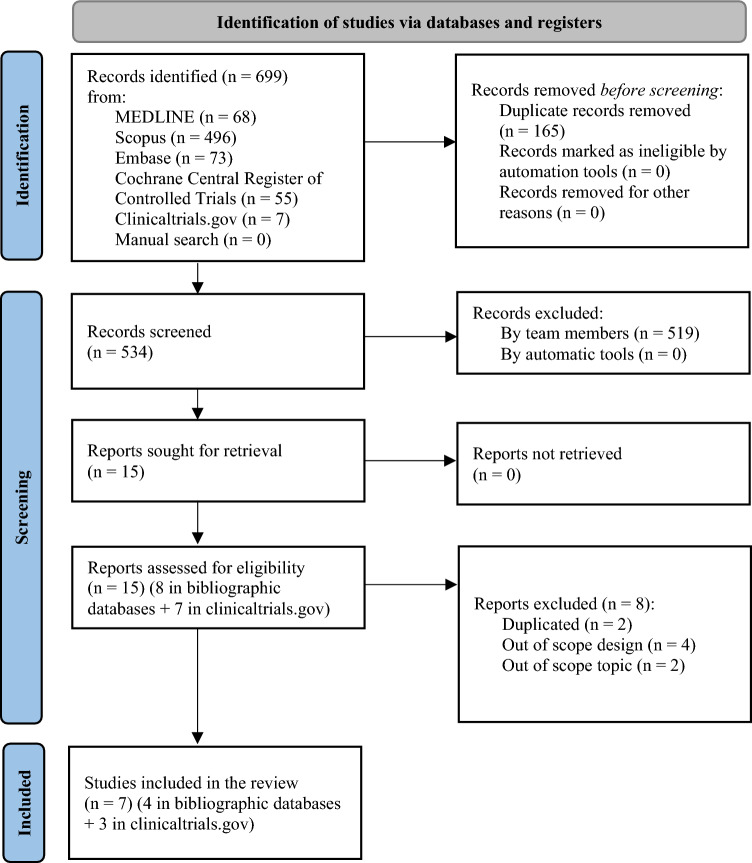
Flow diagram of the scoping review according to PRISMA 2020 for systematic reviews which included searches of databases and registers

The search strategy generated 699 references via bibliographic databases, including 7 references identified from registries (see Fig. 1, flow diagram of the ScR according to PRISMA). From the 699 references, 534 references were screened after excluding duplicates, 15 studies were assessed for eligibility and 8 were further excluded (2 were duplicated [16, 17], 4 had out of scope designs [18–21], and 2 had out of scope topics [22, 23]). The percentage of agreement between reviewers for titles and abstract screening was 97.4%, indicating almost perfect agreement [24]. Consensus was achieved for the 14 studies where the two reviewers disagreed.

Finally, 7 studies (4 articles [4, 5, 25, 26] and 3 registered protocols (clinicaltrials.gov) [27–29]) meet eligibility criteria and were included. The 7 studies included were randomized controlled trials [4, 5, 25–29]. No network meta-analysis, meta-analysis, or systematic reviews were found. No studies by manual search were included.

Six of the 7 studies included IC interventions targeted on community-dwelling older adults [4, 5, 25–28] and one study protocol in nursing home population [29]. The characteristics of the populations varied, with some shared inclusion criteria among studies, such as older age [range ≥ 65 to ≥ 75-year-old], frailty or prefrailty, a decline in at least one IC domain, and ability to communicate and ambulate with/without aids. All interventions in the 7 RCT were based on physical exercise programs: five included multicomponent exercises programs [5, 25, 27–29], from which three [5, 27, 28] applied the Vivifrail intervention [30], an evidence-based multicomponent exercise program which has been recommended as the elective intervention for patients with decline in the IC locomotor domain by the ICOPE guidelines [31], and two applied interventions specifically designed for those trials [25, 29]. One study included an oral supplementation with omega-3 polyunsaturated fatty acids [25], one study included resistance training, aerobic training, or both training combined [4], and one used resistance training, alone or combined with instability devices [26]. No pharmacological interventions were found. The comparator in the control group of the 7 RCT was usual care (i.e., standard of care) [4, 5, 25–29]. The duration of the interventions ranged from 12 [5, 26, 27] to 156 weeks [25]. Additional information is available in Supplementary material Table 3S.

**Fig. 2:**
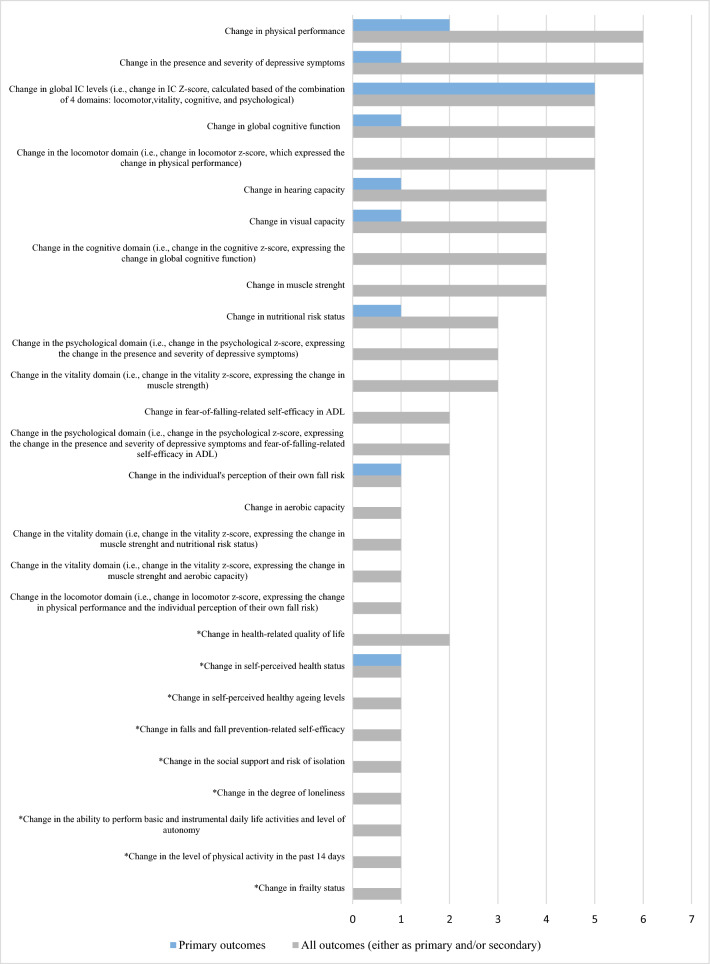
Frequency of the 28 outcomes reported in Intrinsic capacity (IC) intervention studies: 19 outcomes related to IC and its domains and 9 unrelated outcomes (*n* = 7). The * indicates the 9 outcomes unrelated to IC and its domains, reported in IC intervention studies. The number of outcomes unrelated to IC and its domains and the number of measurement tools unrelated to IC and its domains are different (9 and 8, respectively), as the measurement tool for change in frailty status was not explicitly mentioned

The ScR identified 28 outcomes within the 7 RCTs, whose frequency (either as primary and/or secondary) is shown in Fig. 2. Of the 28 outcomes, 19 were related to IC and its domains and 9 were unrelated to IC or any specific domain (e.g., quality of life, etc.).

The most frequently reported primary outcome was the change in IC levels postintervention (5 over 7 studies) [4, 5, 25, 26, 29]. The most frequently reported outcomes (either as primary and/or secondary) were the change in physical performance (6 over 7 studies) [5, 25–29] and the change in depressive symptoms (6 over 7 studies) [5, 25–29]. Additional information is available in Table 2 and Supplementary material Table 3S.

**Table 2:** Summary of findings of the ScR reporting the concept/definition and measurement tools used to assess the Intrinsic capacity domains in the studies (*n* = 7—newest studies first)

First author, year of publication, journal/registry	Locomotor domain	Vitality domain	Cognitive domain	Psychological domain	Sensorial domain
Cavalcante, 2023, Rev. Pesqui. Fisioter	*Physical performance (mobility, gait speed, and static balance)* Measured by a * z*-score of the combination of four tests Timed Up and Go (TUG)(s) Short Physical Performance Battery (SPPB) [0–12] 4-m test (m/s) Timed Chair Stand Test (TCST) (s)	*Muscle strength and aerobic capacity* Measured by a * z*-score of the combination of two tests Handgrip strength (kg) 6-min walking test (6MWT) (m)	*Global and executive cognitive function, i.e., verbal fluency, processing speed and working memory, and immediate and late logical memory* Measured by a * z*-score of the combination of the eight subtest which form the Montreal Cognitive Assessment (MoCA, /30), i.e., sum of words starting with F, A, and S + naming of animals), (codes test, trail test, part B minus part A, and Digit Span, Forward minus Backward), and immediate and late logical memory)	*Depressive symptoms and self-efficacy (fear-of-falling-related self-efficacy in ADL)* Measured by a * z*-score of the combination of two tests Geriatric Depression Scale-15 (GDS-15), [0–15] Fall Efficacy Scale Index [16–64]	Not assessed
Zhang, 2023, Clinicaltrials.gov NCT05891782	*Physical performance (mobility, gait speed, and static balance)* Measured by the Short Physical Performance Battery (SPPB), /12	*Muscle function and nutritional risk status* Measured by two tests Handgrip strength (kg) Mini-Nutritional Assessment full form (MNA, /30)	*Cognitive ability* Measured by the Mini-mental State Examination (MMSE, /30, altered if < 24)	*Presence and severity of depressive symptoms* Measured by the Patient Health Questionnaire-9 (PHQ-9) (/27, having depressive symptoms if ≥ 5)	*Sensory capacity* Measured by the presence of visual distance difficulties, reading difficulties, eyes disease or under treatment, if not, visual acuity chart tests (< 0.8) Measured by the presence of communication difficulties caused by hearing, use of hearing aids or related diseases, if not, whispering test (repeat word correctly)
Gimenez Mestre, 2023, Clinicaltrials.gov NCT05744492	Measured by the Short Physical Performance Battery (SPPB), /12	Measured by two tests Handgrip strength (kg) MNA-SF, /14	Measured by the Montreal Cognitive Assessment (MoCA), /30	Measured by the Geriatric Depression Scale (GDS-15), /15	*Visual and hearing capacity* Measured by the WHO simple eye charts Measured by the HearWHO App, i.e., an automated digit-in-noise test
Blancafort Alias, 2022, Clinicaltrials.gov NCT05249504	*Screening* by the 5-times chair rise > 14 s AND *Diagnosis* by SPPB < 10 OR 4-m gait speed ≤ 0.8 m/s	*Screening* by any nutritional issue from the ICOPE screening tool (loss of appetite OR unintentional weight loss ≥ 3 kg in the last 3 months) AND *Diagnosis* by a MNA-SF < 12 points	*Screening* by any cognitive issue from the ICOPE screening tool (any time or space orientation failure OR not recalling 3 words) AND *Diagnosis* by Mini-Mental State Examination (MMSE, i.e., a Spanish adaptation, Mini Cognitive Examination < 24)	*Screening* by any depressive symptoms from the ICOPE screening tool (sadness, melancholy OR hopelessness or lack of interest or pleasure when doing things) AND Diagnosis by Geriatric Depression Scale GDS-5, ≥ 2	Measured by reporting sight problems AND visual acuity < 6/60 in tumbling E chart. Measured by failing to repeat a minimum of 4 words spelled in a whisper voice with at least one ear AND Light hearing loss in one ear (hearing test audiogram score < 6)
Sánchez-Sánchez, 2022, Age and Ageing	*Locomotion* Measured by the Short Physical Performance Battery (SPPB), /12	*The vitality domain* Measured by the handgrip strength (kg)	*The global cognition* Measured by the Montreal Cognitive Assessment (MoCA), /30	*Psychological domain* Measured by the Geriatric Depression Scale-15 (GDS-15), /15	*The sensory domain* Measured by a score based on the presence of self-reported reduced visual acuity (yes = 1, no = 0), and hypoacusia (yes = 1, no = 0) (range 0–2)
Giudici, 2020, Maturitas	*Locomotion, assessing mobility aspects* Measured by the Short Physical Performance Battery (SPPB), /12	*Physiological reserves assessment* Measured by the handgrip strength (kg)	*Cognition (episodic memory, timed executive function, and global cognition)* Measured by four tests The ten orientation items of the MMSE Digit Symbol Substitution test Free and total recall of Free and Cued Selective Reminding test Category Naming test	*Presence and severity of depressive symptoms* Measured by the Geriatric Depression Scale (GDS-15), /15	Not assessed
Huang, 2020, JAMDA	*Locomotion* Measured by three tests One-leg stand test (OLS) 5-m walking speed test (s) Timed Chair Stand test (TCST) (s)	*Vitality* Measured by the handgrip strength (kg)	*Cognition (immediate and delayed memory, verbal fluency, visuospatial memory, processing speed, and executive function)* Measured by seven tests Logical Memory I from Wechsler Memory Scale-Revised Logical Memory II from Wechsler Memory Scale-Revised Category fluency test and letter fluency test from MMSE Pentagon copying test from MMSE Digit Symbol test from Wechsler Adult Intelligence Scale-III Trail Making Test-part A (s) Trail Making Test-part B (s)	*Psychological function* Measured by a * z*-score of the combination of two tests Geriatric Depression Scale (GDS-15), /15 Generalized Anxiety Disorder-7 scale (GAD-7), /7	Not assessed

Fifty-five measurement tools were identified (47 related to IC and its domains and 8 unrelated). The most frequently reported tool was an IC *Z*-score, aiming to reflect the IC as a global construct, which represented the distance to the studied population to the mean, expressed in standard deviations, calculated based on the individual *z*-scores of the individual domains [4, 5, 25, 26, 29]. The IC *Z*-score was calculated based on the individual *z*-scores of 4 domains’ *z*-scores: locomotor, vitality, cognitive, and psychological (5 over 7 studies) [4, 5, 25, 26, 29]. Additionally, one study protocol planned to take the sensorial domain into consideration to calculate the IC *Z*-score [29].

**Fig. 3:**
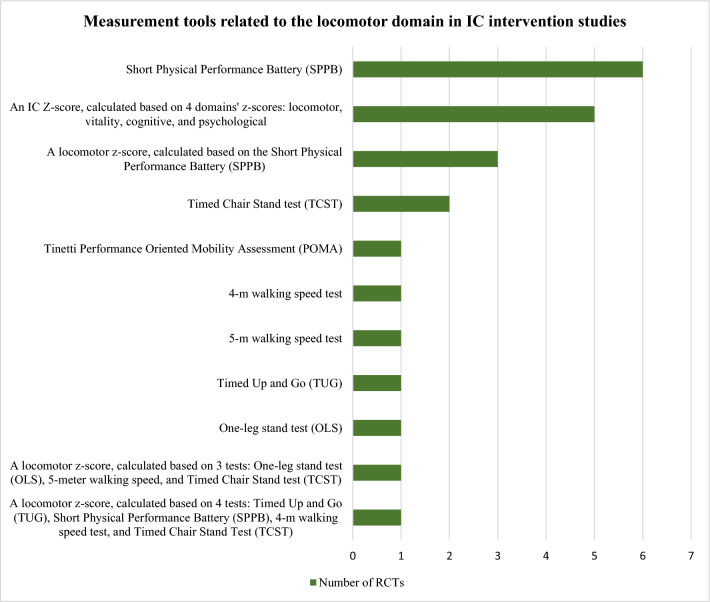
Frequency of the IC *Z*-score and the ten measurement tools related to the locomotor domain in IC intervention studies (*n* = 7). For scoping review purposes, the IC *Z*-score, the domains’ *z*-scores, and the tests have been considered as “measurement tools’’ (i.e., “measurement tools “ equals “assessment tools”, “tools”, or “tests”)

**Fig. 4:**
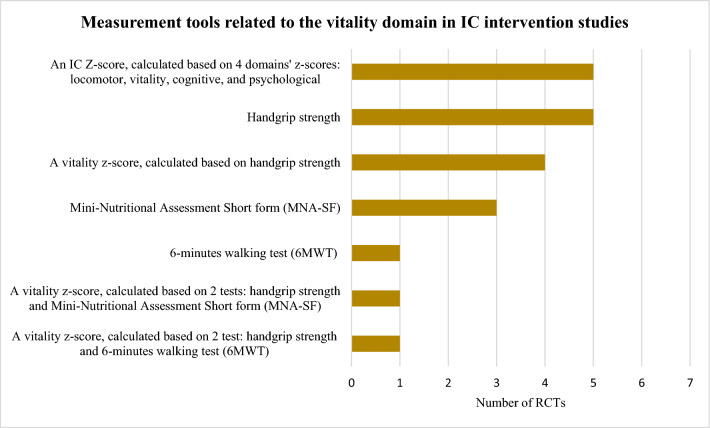
Frequency of the IC *Z*-score and the six measurement tools related to the vitality domain in IC intervention studies (*n* = 7). For scoping review purposes, the IC *Z*-score, the domains’ *z*-scores, and the tests have been considered as “measurement tools’’ (i.e., “measurement tools” equals “assessment tools”, “tools”, or “tests”)

**Fig. 5:**
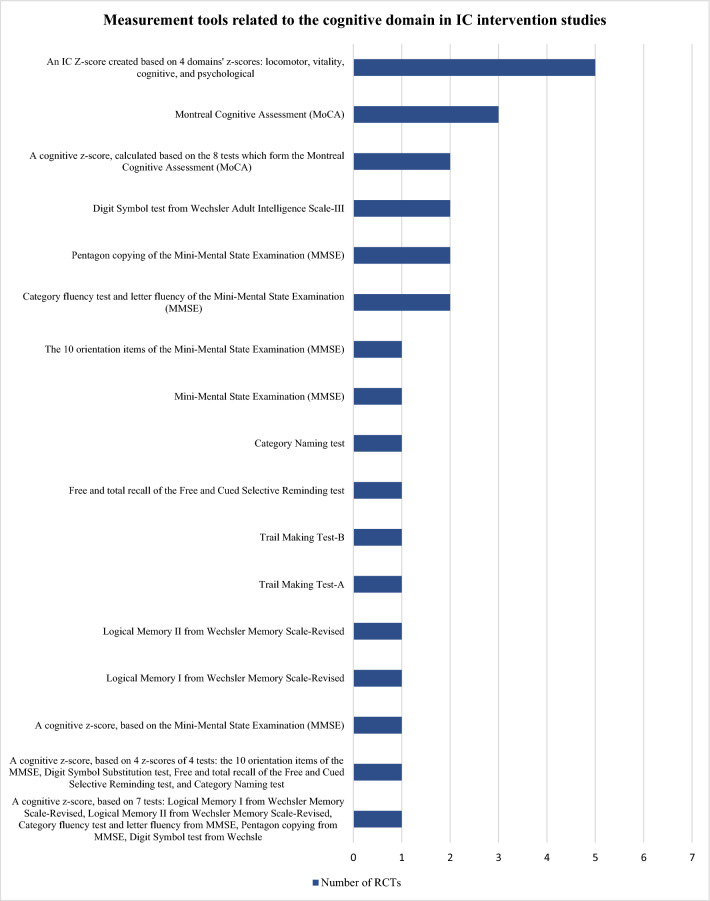
Frequency of the Intrinsic capacity (IC) *Z*-score and the 16 measurement tools related to the cognitive domain in IC intervention studies (*n* = 7). For scoping review purposes, the IC *Z*-score, the domains’ *z*-scores, and the tests have been considered as “measurement tools’’ (i.e., “measurement tools” equals “assessment tools”, “tools”, or “tests”)

**Fig. 6:**
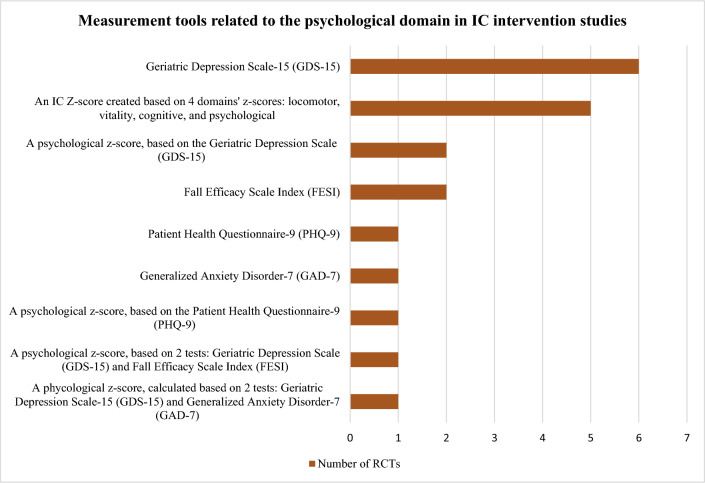
Frequency of the Intrinsic capacity (IC) *Z*-score and the eight measurement tools related to the psychological domain in IC intervention studies (*n* = 7). For scoping review purposes, the IC *Z*-score, the domains’ *z*-scores, and the tests have been considered as “measurement tools” (i.e., “measurement tools” equals “assessment tools”, “tools”, or “tests”)

**Fig. 7:**
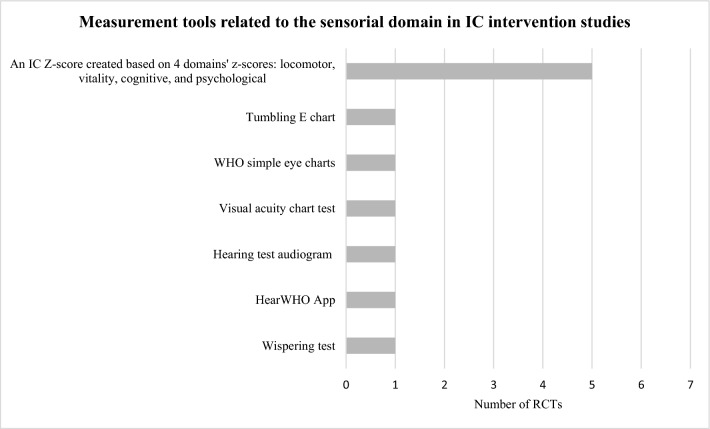
Frequency of the Intrinsic capacity (IC) *Z*-score and the six measurement tools related with the sensorial domain in IC intervention studies (*n* = 7). For scoping review purposes, the IC *Z*-score, the domains’ *z*-scores, and the tests have been considered as “measurement tools” (i.e., “measurement tools” equals “assessment tools”, “tools”, or “tests”). The IC *Z*-score has been included in this figure for figure drafting purposes, due to the low frequencies of these tools in the RCTs

**Fig. 8:**
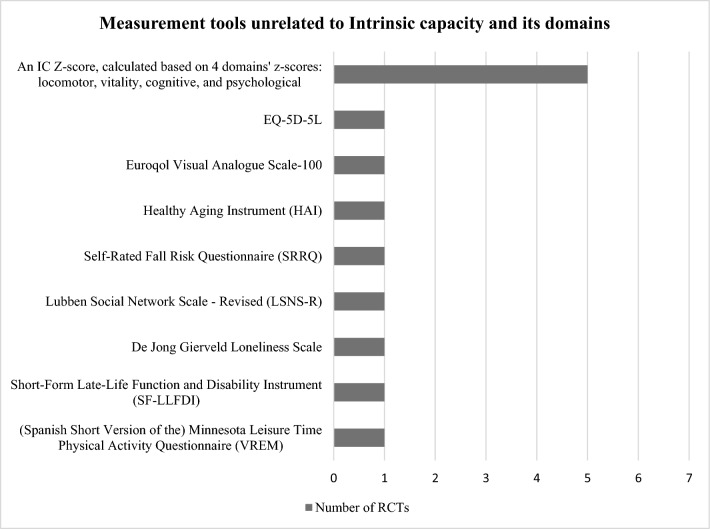
Frequency of the 8 measurement tools, unrelated to Intrinsic capacity (IC) and its domains, reported in IC intervention studies (*n* = 7). For scoping review purposes, the IC *Z*-score, the domains’ *z*-scores, and the tests have been considered as “measurement tools” (i.e., “measurement tools” equals “assessment tools”, “tools”, or “tests”). The IC *Z*-score has been included in this figure for figure drafting purposes, due to the low frequencies of these tools in the RCTs. The number of outcomes unrelated to IC and its domains and the number of measurement tools unrelated to IC and its domains are different (9 and 8, respectively), as the measurement tool for change in frailty status was not explicitly mentioned

The tools identified were used to construct the domains’ *z*-scores and/or assess the effect of the interventions and differed widely among studies: 10 locomotor related, 6 vitality related, 16 cognitive related, 8 psychological related, 6 sensorial-related tools, and 8 tools unrelated to IC. Figures 3, 4, 5, 6 and 7 show the frequency of the IC *Z*-score, the domains’ *z*-scores, and the measurement tools related to IC and its five domains reported in IC intervention studies. A large heterogenicity was found in the construction of the domains’ *z*-score, i.e., even if 5 studies used the same 4 domains to construct the IC *Z*-score, in turn, the domains’ *z*-scores were constructed based on different tools which did not overlap among studies [4, 5, 25, 26, 29]. Figure 8 shows the frequency of the 8 tools unrelated to IC and its domains.

None of the RCT stablished or reported the clinically meaningful significant change or the substantial change of the tools. None of the trials utilized biochemical markers. The vast heterogeneity (28 outcomes and 55 tools within 7 studies) highlighted the urgent need of a COS for IC interventions.

## Discussion

This is the first scoping review that identified the outcomes and measurement tools investigated in RCT aiming at the management of IC in midlife and beyond, using any type of intervention, and found 7 IC intervention studies (4 articles and 3 registered protocols) which met eligibility criteria. Twenty-eight outcomes were identified within the 7 RCTs, from which 19 outcomes were related to IC and its domains and 9 were unrelated to IC or any specific domain (e.g., quality of life, etc.). The most reported primary outcome was the change in IC levels postintervention [4, 5, 25, 26, 29], and the most reported outcomes (either as primary and/or secondary) were the change in physical performance and the change in depressive symptoms [5, 25–29].

From the 55 measurement tools identified, the most frequently reported was an IC *Z*-score, calculated by 4 domains’ *z*-scores: locomotor, vitality, cognitive, and psychological [4, 5, 25, 26, 29]. Additionally, one study protocol planned to take the sensorial domain into consideration to calculate the IC *Z*-score [29]. Six RCTs used the SPPB as measurement tool for the locomotor domain, which is aligned with updated recommendations by the WHO locomotor capacity working group [32]. The locomotor capacity has been recently defined as “a state (static or dynamic over time) of the musculoskeletal system that encompasses endurance, balance, muscle strength, muscle function, muscle power, and a joint function of the body” [32]. Moreover, as part of this WHO initiative, a ScR is undergoing, aimed to shed light about the measurement tools for this domain, which may be helpful as supportive material for the decision making about this domain in upcoming steps of the COS process. The handgrip strength and/or the MNA-SF were the tools most frequently employed for the vitality domain; to the authors knowledge, the recommended tools for this domain are still under debate among WHO and others [33, 34]. The MMSE and the MoCA where the most reported tools for the cognitive domain, the GDS-15 was the most reported for the psychological domain, and a large heterogenicity was observed in the assessment tools for the sensorial domain, which was rarely included in the IC *Z*-score calculation [29]. Specific initiatives about these domains are still unavailable and urgently needed. The tools used to construct the domains’ *z*-scores differed among trials and the domains’ *z*-scores did not overlap. These findings are consistent with a previous narrative review, which explored the tools used for quantitative measurement of IC and its domains, in any study type. The review identified ten studies and found low concordance among the tools measuring each domain. The main gaps identified were that most of studies undertook the analysis of the individual domains rather than the IC as a construct, the lack of consensus in the tools for domain assessment, and the limitations derived from those scores which depend on the distribution of the study sample [34].

Some strengths and limitations of the ScR should be acknowledged. First, one of the major strengths is the highest methodological quality, including a systematic search following PRISMA-ScR, in four different bibliographic databases and registered studies [15]. Second, the ScR is timely, because developing a COS is ideally done after evidence is available but before many intervention studies have been conducted, to ensure that future trials can adhere to the COS. So, the review addresses a major research gap, as no other COS initiative in IC had been launched, and such initiatives are scarce in Geriatric Medicine, despite being urgently needed. Finally, the comprehensive listing and inclusion of the IC *Z*-score, the domains’ *z*-scores, and the tools is relevant and ensures the optimal practicality for the future COS. Given that the COS is still under development, the specific number of tools required to measure each domain is unknown at this stage. Therefore, listing the intermediate step, i.e., the domains’ *z*-scores ensure coherency in the outcomes, tools, and anticipate the consistency in the intermediate steps required for IC *Z*-score calculation. Since the COS in IC intervention studies is intended to be of interest to a large clinical and scientific community, Supplementary material, Fig. 1S offers clarifying examples for better understanding.

Nevertheless, the ScR has two limitations. First, the review was focused on those interventions where IC was considered as a whole construct and with the highest quality design only (i.e., RCT). This may have left aside those interventions aimed at improving physiological or pathological aspects of the ageing process in a particular domain or condition alone (e.g., RCT aimed at improving memory, physical performance, frailty status, etc.) or with a different study design. This could be considered a minor limitation because including studies with inadequate designs may have retrieved a larger number of studies, but may have decreased the quality of the ScR and incorporated bias and methodological limitations due to flaws in the study designs. Secondly, the terminology and categorization used for ScR purposes may differ from those reported in the original studies, where the tools are reported, but not the outcomes they referred to, and where the domains’ *z*-scores calculation are implied but unstated. This is a minor limitation, as the categorization was needed for ScR synthesis purposes, was taken by consensus, and preserved the key aspects of the original studies.

Future IC trials may need to align their outcomes and tools with existing ones and the COS, to streamline data comparability, enable direct and indirect comparisons among studies (network-meta analysis), reduce duplication of efforts, and maximize efficiency in promoting better ageing processes. This is particularly important in older people, a population which is frequently underrepresented in clinical trials, and where recruitment and retention are frequently challenging. The development of the COS for IC intervention studies may be relevant for institutions aimed to drug development and regulation, such as the European Medicines Agency (EMA) or the United States Food and Drug Administration (FDA), as COS boost the evidence about treatment effectiveness, promote patient-centered approaches, and support regulatory decision-making. These benefits for a large variety of stakeholders also may explain the recently developed COS for malnutrition [8], sarcopenia [9, 10], and frailty [11], aimed at covering unmet needs in these tree conditions, and which have been developed following similar procedures. Moreover, it is crucial to emphasize the recent advances and potential role of biological markers [9, 35], which have been underused in IC trials to date but hold promise for playing a crucial role in future trials.

*Further steps* of the COS for IC studies are: (1) A modified 2-round Delphi study, to rank the outcomes and assessment tools by a group of international experts, both clinical and research professionals; (2) As patients’ values and preferences should be taken into consideration, the insight from adult population in midlife and beyond (either healthy participants and patients with declines in IC) will be collected from 10 direct interviews (qualitative research); (3) Consensus meetings among experts will be conducted afterwards; (4) Finally, it is expected that the COS for IC studies will be reported following the COS-STAR recommendations.

## Conclusions

This is the first ScR that identified the outcomes and measurement tools investigated in RCT aiming at the management of IC in older people, using any type of intervention. The review has a high methodological quality, follows PRISMA-ScR, and is part of a larger process of developing a COS for IC intervention studies, which will involve international experts, key stakeholders, and the insight of the target population.

The vast heterogeneity (28 outcomes and 55 tools within 7 studies) confirmed the major research gap and the urgent need of developing a COS for IC intervention studies. The outcomes and measurement tools identified by the review provide essential evidence to guide further steps of the COS development process, including a modified 2-round Delphi study, quality research interviews in the target population, consensus meetings, and COS final reporting following COS-STAR guidelines. A COS for IC intervention studies can be helpful to harmonize outcomes and measurement tools, enhance transparency in IC trials, facilitate effective research through comparisons and network-meta-analysis among studies, and ultimately guide clinical and Public Health Actions in midlife and beyond, within the framework of the Decade of Healthy Aging.

### Supplementary Information

Below is the link to the electronic supplementary material.

Supplementary file1 (DOCX 70 KB)

Supplementary file2 (DOCX 89 KB)
