# Supplementary material for: Towards a core outcome set (COS) for intrinsic capacity (IC) intervention studies in adults in midlife and beyond: a scoping review to identify frequently used outcomes and measurement tools
Source: Aging Clin Exp Res. 2024 Mar 5;36(1):54. doi: 10.1007/s40520-023-02681-8 (PMC10914863; doi:10.1007/s40520-023-02681-8)
Supplement: Supplementary file 1 — Supplementary file1 (DOCX 70 KB) [file 40520_2023_2681_MOESM1_ESM.docx]

**
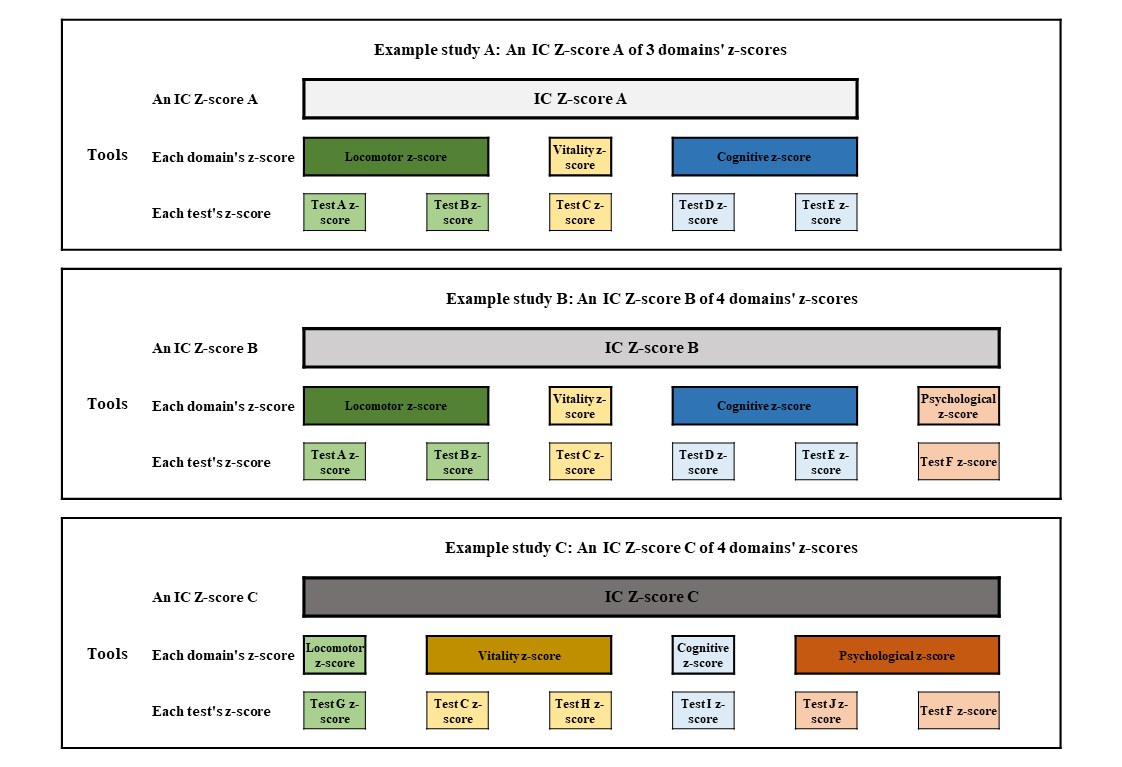
Figure 1S**. Clarification of terminology in the scoping review: Intrinsic Capacity (IC) Z-score, domains' z-scores, and the tests.

**For ScR purposes, the IC Z-score, the domains’ z-scores, and the tests have been considered as “measurement tools’’** (i.e., equal “assessment tools”, “tools” or “tests”)**.**

The IC Z-score mentioned in the ScR and some of the trials refers to a statistical measurement of IC levels. It aims to reflect the IC as a global construct, representing the distance of the studied population from the mean, expressed in standard deviations, and calculated based on the domains’ z-scores. The Z-score was considered as a tool in this ScR. This decision was made because the outcome of these trials was defined as “the change in global IC postintervention’’, and IC was measured by the Z-score. Thus, the IC Z-score served as the IC measurement tool.

In studies using the IC Z-score as the tool, the calculation necessarily involves the domains' z-scores as an implicit, mandatory intermediate step. The rationale for including the intermediate step (domains' z-scores) among the measurement tools was to provide a detailed account of all elements required to construct the IC Z-score. This level of detail was considered relevant for ScR and future COS purposes, especially since the future COS might include multiple tests to assess each domain. The combination of these tests in different ways demands that the construction of the domains' z-scores be explicitly listed in the ScR and taken into consideration during COS development.

**As *explanatory examples,*** three studies (Studies A, B, and C) which use the Intrinsic capacity (IC) Z-score as a tool to reflect the IC levels have been represented above. In the three studies (*first column on the left*) it is represented the three levels of “tools”: each IC Z-score is calculated based on the domains' z-scores, and in turn, each domain's z-score is based on the z-score of one or more tests.

In those domains which are based only in one test’s z-score, the z-score of the test equals the z-score of the domain. In those domains which are based on more than one test, the z-score of the domain is calculated based on the z-scores of multiple tests.

**Study A and Study B.** Both studies, A and B, use the same tests to construct most of their domain z-scores. However, Study B employs a different number of domains to calculate the IC Z-score. Consequently, comparisons of the IC Z-scores between Study A and Study B are not possible due to their calculation based on a different number of domains.

**Study B and C:** Studies B and C both employ the same number of domains to construct the IC Z-score. However, there is a difference in the tests used to construct each domain's z-score between the two studies. For instance, in Study B, the locomotor z-score is constructed using the combination of Test A's z-score and Test B's z-score, whereas in Study C, the locomotor domain is based solely on Test G's z-score. Due to these differences in test selection, direct comparisons of the IC Z-scores and domain z-scores between Studies B and C cannot be made. In Study C, the locomotor domain relies solely on Test G, making the z-score of Test G equivalent to the z-score of the locomotor domain. On the other hand, the vitality domain in Study C is based on multiple tests (Test C and Test H), and its z-score is calculated from the z-scores of Test C and Test H.

In summary, the following elements have been considered and listed under the term “tools”:

- IC Z-score (calculated based on the domains' z-scores).
- Domains' z-scores (e.g., locomotor z-score, vitality z-score, etc.).
- The tools to construct the domains' z-scores (these may be equal to the domain z-score if only one test is used or different if multiple tools are used within a domain).
