# Supplementary material for: Towards a core outcome set (COS) for intrinsic capacity (IC) intervention studies in adults in midlife and beyond: a scoping review to identify frequently used outcomes and measurement tools
Source: Aging Clin Exp Res. 2024 Mar 5;36(1):54. doi: 10.1007/s40520-023-02681-8 (PMC10914863; doi:10.1007/s40520-023-02681-8)
Supplement: Supplementary file 2 — Supplementary file2 (DOCX 89 KB) [file 40520_2023_2681_MOESM2_ESM.docx]

**Supplementary material, Table 1S**: Preferred Reporting Items for Systematic reviews and Meta-Analyses extension for Scoping Reviews (PRISMA-ScR) Checklist. Towards a Core Outcome Set (COS) for Intrinsic Capacity (IC) intervention studies in older people: a scoping review to identify frequently used outcomes and measurement tools.

| **SECTION** | **ITEM** | **PRISMA-ScR CHECKLIST ITEM** | **REPORTED ON PAGE #** |
| --- | --- | --- | --- |
| **TITLE** | | | |
| Title | 1 | Identify the report as a scoping review. | 1 |
| **ABSTRACT** | | | |
| Structured summary | 2 | Provide a structured summary that includes (as applicable): background, objectives, eligibility criteria, sources of evidence, charting methods, results, and conclusions that relate to the review questions and objectives. | 1 |
| **INTRODUCTION** | | | |
| Rationale | 3 | Describe the rationale for the review in the context of what is already known. Explain why the review questions/objectives lend themselves to a scoping review approach. | 2 |
| Objectives | 4 | Provide an explicit statement of the questions and objectives being addressed with reference to their key elements (e.g., population or participants, concepts, and context) or other relevant key elements used to conceptualize the review questions and/or objectives. | 3-4 |
| **METHODS** | | | |
| Protocol and registration | 5 | Indicate whether a review protocol exists; state if and where it can be accessed (e.g., a Web address); and if available, provide registration information, including the registration number. | 5 |
| Eligibility criteria | 6 | Specify characteristics of the sources of evidence used as eligibility criteria (e.g., years considered, language, and publication status), and provide a rationale. | 5 and table 1 |
| Information sources* | 7 | Describe all information sources in the search (e.g., databases with dates of coverage and contact with authors to identify additional sources), as well as the date the most recent search was executed. | 5 and table 2S |
| Search | 8 | Present the full electronic search strategy for at least 1 database, including any limits used, such that it could be repeated. | Table 2S, search for 4 databases |
| Selection of sources of evidence† | 9 | State the process for selecting sources of evidence (i.e., screening and eligibility) included in the scoping review. | 5 and 6 |
| Data charting process‡ | 10 | Describe the methods of charting data from the included sources of evidence (e.g., calibrated forms or forms that have been tested by the team before their use, and whether data charting was done independently or in duplicate) and any processes for obtaining and confirming data from investigators. | 6 |
| Data items | 11 | List and define all variables for which data were sought and any assumptions and simplifications made. | 5 and 6 |
| Critical appraisal of individual sources of evidence§ | 12 | If done, provide a rationale for conducting a critical appraisal of included sources of evidence; describe the methods used and how this information was used in any data synthesis (if appropriate). | Not applicable |
| Synthesis of results | 13 | Describe the methods of handling and summarizing the data that were charted. | 6 |
| **RESULTS** | | | |
| Selection of sources of evidence | 14 | Give numbers of sources of evidence screened, assessed for eligibility, and included in the review, with reasons for exclusions at each stage, ideally using a flow diagram. | 7 and figure 1 |
| Characteristics of sources of evidence | 15 | For each source of evidence, present characteristics for which data were charted and provide the citations. | 7 |
| Critical appraisal within sources of evidence | 16 | If done, present data on critical appraisal of included sources of evidence (see item 12). | Not applicable |
| Results of individual sources of evidence | 17 | For each included source of evidence, present the relevant data that were charted that relate to the review questions and objectives. | 7 and 8, tables 2, 3, and supplementary table 3S |
| Synthesis of results | 18 | Summarize and/or present the charting results as they relate to the review questions and objectives. | 7 and 8 |
| **DISCUSSION** | | | |
| Summary of evidence | 19 | Summarize the main results (including an overview of concepts, themes, and types of evidence available), link to the review questions and objectives, and consider the relevance to key groups. | 9 |
| Limitations | 20 | Discuss the limitations of the scoping review process. | 9 |
| Conclusions | 21 | Provide a general interpretation of the results with respect to the review questions and objectives, as well as potential implications and/or next steps. | 10 |
| **FUNDING** | | | |
| Funding | 22 | Describe sources of funding for the included sources of evidence, as well as sources of funding for the scoping review. Describe the role of the funders of the scoping review. | 9 |

**Supplementary material, Table 2S.** Search strategy (from inception to 16/06/2023).

**______________________________________________________________________**

**Medline** (Ovid)

-------------------------------------------------------------------------------------------------------

1 (intrinsic adj1 capacit*).ti,ab,kf. (1017)

2 Randomized Controlled Trial/ (590348)

3 randomi*.ti,ab,kf. (797001)

4 RCT*.ti,ab,kf. (73632)

5 Random Allocation/ (106917)

6 Double-Blind Method/ (174803)

7 single-blind method/ (32611)

8 ((singl* or doubl* or trip* or trebl*) adj2 (blind* or mask* or conceal*)).ti,ab,kf. (197259)

9 exp clinical trial/ (967252)

10 Controlled Clinical Trial/ (95252)

11 ((clinic* or interventi*) adj4 (stud* or trial*)).ti,ab,kf. (1031958)

12 or/2-11 (2187928)

13 1 and 12 (65)

**Scopus**

----------------------------------------------------------------------------------------------------------

( ( TITLE-ABS-KEY ( randomi* ) ) OR ( TITLE-ABS-KEY ( rct ) ) OR ( TITLE-ABS-KEY ( "randomized controlled trial" ) ) OR ( TITLE-ABS-KEY ( ( ( singl* OR doubl* OR trip* OR trebl* ) W/2 ( blind* OR mask* OR conceal* ) ) ) ) OR ( TITLE-ABS-KEY ( ( ( clinic* OR interventi* OR controlled ) W/2 ( study OR trial ) ) ) ) ) AND ( TITLE-ABS-KEY ( intrinsic W/2 capacit* ) ) AND ( LIMIT-TO ( PUBYEAR , 2016 ) OR LIMIT-TO ( PUBYEAR , 2017 ) OR LIMIT-TO ( PUBYEAR , 2018 ) OR LIMIT-TO ( PUBYEAR , 2019 ) OR LIMIT-TO ( PUBYEAR , 2020 ) OR LIMIT-TO ( PUBYEAR , 2021 ) OR LIMIT-TO ( PUBYEAR , 2022 ) OR LIMIT-TO ( PUBYEAR , 2023 ) ) 462 results

**Embase**

----------------------------------------------------------------------------------------------------------

**No.**

**Query**

**Results**

**1,734,801**

**#14**

**#3** OR **#4** OR **#5** OR **#6** OR **#7** OR **#8** OR **#9** OR **#10** OR **#11** OR **#12**

**1,100**

**#13**

**#1** OR **#2**

**2,437**

**#12**

**'controlled intervention'**:ab,kw,ti

**230,571**

**#11**

**'controlled trial'**:ab,kw,ti

**72,079**

**#10**

**'controlled study'**:ab,kw,ti

**6,883**

**#9**

**'clinical intervention'**:ab,kw,ti

**90,650**

**#8**

**'clinical study'**:ab,kw,ti

**292,411**

**#7**

**'clinical trial'**:ab,kw,ti

**109,454**

**#6**

**rct***:ab,kw,ti

**1,141,033**

**#5**

**randomi***:ab,kw,ti

**248,875**

**#4**

**'randomized controlled trial (topic)'**

**1,022,991**

**#3**

**'randomized controlled trial'**

**1,100**

**#2**

**'intrinsic capacity'**

**15**

**#1**

**'intrinsic capacity'**/exp

**EBM Reviews -** **Cochrane Central Register of Controlled Trials**

----------------------------------------------------------------------------------------------------------

1 (intrinsic adj1 capacit*).ti,ab,kf. (31)

2 Randomized Controlled Trial/ (25743)

3 randomi*.ti,ab,kf. (952168)

4 RCT*.ti,ab,kf. (37367)

5 Random Allocation/ (23359)

6 Double-Blind Method/ (154027)

7 single-blind method/ (24370)

8 ((singl* or doubl* or trip* or trebl*) adj2 (blind* or mask* or conceal*)).ti,ab,kf. (312779)

9 exp clinical trial/ (45358)

10 Controlled Clinical Trial/ (17157)

11 ((clinic* or interventi*) adj4 (stud* or trial*)).ti,ab,kf. (359486)

12 or/2-11 (1157496)

13 1 and 12 (27)

| **First author, year of publication, journal/registry** | **Primary outcome(s) as quoted by authors** | **Primary outcomes rephrased for ScR purposes**  *Individual items in this column correspond directly to adjacent items in the column on the left and the right. The items related to each domain are grouped. “Change” relates to the change between pre and post-intervention* | **Measurement tools for the primary outcome** | **Secondary outcome(s) as quoted by authors** | **Secondary outcomes rephrased for ScR purposes** *Individual items in this column correspond directly to adjacent items in the column on the left and the right. The items related to each domain are grouped. “Change” relates to the change between pre and post-intervention* | **Measurement tools for the secondary outcome** | **Comments** *Two columns have been created: ''outcomes as quoted by authors'' and ''outcomes rephrased for ScR purposes'', for clarity, due to the language gap between the original texts and the rephrased outcomes for review purposes* |  |
| --- | --- | --- | --- | --- | --- | --- | --- | --- |
| Cavalcante, 2023, Rev. Pesqui. Fisioter. | “Difference (improvement) in overall levels of IC” | The change in global IC levels (i.e., change in an IC Z-score of the combination of 4 domains: locomotor,vitality, cognitive, and psychological).  Calculation: The outcome was a unique raw value, i.e., a continuous variable with a 95% confidence interval, calculated by the difference between two different IC global Z-scores, constructed with the same variables, at two different time points (i.e., pre and post-intervention), and which represents the distance to the mean of the sample, after the intervention | An IC Z-score, calculated based on 4 domains' z-scores: locomotor, vitality, cognitive, and psychological   Locomotor, vitality, and cognitive domains were identically weighted, because higher scores indicated improvement on this domain. The psychosocial domain was negatively weighted, because higher scores indicated a deterioration.   Note: Missing values were treated by ''Last observation carried forward'', i.e., missing follow-up visit values were imputed as subject's previously observed value (limitation). | Secondary outcome are not explicitely quoted in the text; but the change in the z-scores for each of the 4 domains are reported separately after the primary outcome (results section and tables). | Change of the locomotor domain (i.e, change in the locomotor z-score, which expressed the change in physical performance pre and post-intervention). | Locomotor z-score, calculated based on 4 tests:   - Timed Up and Go (TUG) - Short Physical Performance Battery (SPPB) - 4-m test (m/s) - Timed Chair Stand Test (TCST) | The evolution/change of the IC Z-score is the outcome, i.e., a unique raw value which shows the difference between two different IC Z-scores calculated with the same variables, in the same population, at two different time points (i.e., a continuous variable with a 95% confidence interval), and shows the distance fom the mean measured in SD is the study sample after the intervention. This variable was calculated by the difference between the Z-score pre-intervention and the Z-score post-intervention.   Both pre and post IC Z-scores were equally constructed, based on the individual z-scores for each domain. The Z-score was the tool to depict the IC construct. |  |
|  |  |  |  |  |  |  |  |  |
|  |  |  |  |  | Change in the vitality domain, i.e., change in the vitality z-score, which expressed the change in:   - Muscle strength - Aerobic capacity | Vitality z-score, calculated based on 2 test:   - Handgrip strength and 6-minutes walking test (kg) - 6-minutes walking test (6MWT) (m) |  |  |
|  |  |  |  |  |  |  |  |  |
|  |  |  |  |  |  |  |  |  |
|  |  |  |  |  |  |  |  |  |
|  |  |  |  |  | Change in the cognitive domain, i.e, change in the vitality z-score, which expressed the change in global cognitive function (included visuoespatial and executive function, naming, memory, language, abstraction, delayed recall, and orientation) | Cognitive z-score, calculated based on the 8 components/test of the Montreal Cognitive Assessment (MoCA), /30   - Total MoCA score - Visuoespatial/executive - Naming animals - Attention - Language - Abstraction - Delayed recall - Orientation |  |  |
|  |  |  |  |  |  |  |  |  |
|  |  |  |  |  | Change in the psychological domain, i.e, change in psychological z-score, which expressed the change in:   - The presence and severity of depressive symptoms - Fear-of-falling-related self-efficacy in ADL | Psychological z-score, created based on 2 tests:   - Geriatric Depression Scale (GDS-15) - Fall Efficacy Scale Index (FESI) |  |  |
|  |  |  |  |  |  |  |  |  |
|  |  |  |  |  |  |  |  |  |
|  |  |  |  |  |  |  |  |  |

**Supplementary material, Table 3S** (***part 1/6***)**:** Summary of findings, primary and secondary outcomes, and measurement tools (n = 7 - newest studies first).

**Table 3.** Summary of findings, primary and secondary outcomes, and assessment tools (n = 7 - newest studies first -) *(****part 1/6****).*

| **First author, year of publication, journal/registry**  **Supplementary material, Table 3S *(part 2/6, continuation****)***:** Summary of findings, primary and secondary outcomes, and measurement tools (n = 7 - newest studies first). | **Primary outcome(s) as quoted by authors** | **Primary outcomes rephrased for ScR purposes**  *Individual items in this column correspond directly to adjacent items in the column on the left and the right. The items related to each domain are grouped. “Change” relates to the change between pre and post-intervention* | **Measurement tools for the primary outcome** | **Secondary outcome(s) as quoted by authors** | **Secondary outcomes rephrased for ScR purposes** *Individual items in this column correspond directly to adjacent items in the column on the left and the right. The items related to each domain are grouped. “Change” relates to the change between pre and post-intervention* | **Measurement tools for the secondary outcome** | **Comments** *Two columns have been created: ''outcomes as quoted by authors'' and ''outcomes rephrased for ScR purposes'', for clarity, due to the language gap between the original texts and the rephrased outcomes for review purposes* |
| --- | --- | --- | --- | --- | --- | --- | --- |
| Zhang, 2023, Clinicaltrials.gov NCT05891782 | “An outcome list”, quoting: | The change in global IC levels (i.e., change in an IC Z-score of the combination of 5 domains: locomotor,vitality, cognitive, psychological, and sensorial). | An IC Z-score, calculated based on 5 domains' z-scores: locomotor, vitality, cognitive, psychological, and sensorial is planned | “An outcome list”, quoting: | Change in one or more of the items listed in the secondary outcome list, which includes physical performance, muscle strength, falls and fall-prevention-related self-efficacy, and self-perceived healthy aging. *See comment.* | **-** | None |
|  | “Short Physical Performance Battery (SPPB), includes balance test, 4-m walking speed test, and chair stand test” | Change in physical performance | A locomotor z-score, calculated based on the Short Physical Performance Battery (SPPB), /12. | “The Tinetti Performance Oriented Mobility Assessment (POMA)” | Change in physical performance | Tinetti Performance Oriented Mobility Assessment (POMA), /16 |  |
|  |  |  |  |  |  |  |  |
|  | “Self-Rated Fall Risk Questionnaire (SRRQ), indicating ''a risk of falling” | Change in the individual's perception of their own fall risk | Self-Rated Fall Risk Questionnaire (SRRQ), /14 points, ≥4 score indicates risk of falling. | “Nursing Home Falls Self-Efficacy Scale (NHFSS), the higher the score, the less fear of falling and higher self-efficacy” | Change in fear-of-falling-related self-efficacy in ADL | Nursing Home Falls Self-Efficacy Scale (NHFSS), a version of the Fall Efficacy Scale Index (FESI) adapted for nursing homes |  |
|  | Mini-Nutritional Assessment (MNA)-full form, “to assess nutritional status in the older people (good nutritional status/at risk/malnutrition”) | Change in nutritional risk status | A vitality z-score, calculated based on the MNA-FF (which may be combined with handgrip strenght, listed as secondary outcome). | Handgrip strength | Change in muscle strength | Handgrip strength (kg) |  |
|  |  |  |  |  |  |  |  |
|  | Mini-Mental State Examination (MMSE), “the most influential cognitive ability assessment tool” | Change in cognitive function, i.e., orientation, registration, attention and calculation, recall, language, and visual construction. | A cognitive z-score, calculated based on the Mini-Mental State Examination (MMSE), altered if ,24. | “Healthy Aging Instrument (HAI), higher scores indicating better levels of healthy aging” | Change in self-perceived healthy aging levels based on individual’s perception of oneself healthy ageing, unrelated to the presence or absence of diseases | Healthy Aging Instrument (HAI, /175) |  |
|  |  |  |  |  |  |  |  |
|  | Patient Health Questionnaire-9 (PHQ-9), “having depressive symptoms” | Change in the presence and grading of depression (i.e., depressed mood, anhedonia (loss of interest/pleasure), sleep disturbances, changes in appetite/weight, fatigue, feelings of worthlessness/guilt, difficulty concentrating, psychomotor agitation or retardation, and suicidal thoughts) | A psychological z-score, calculated based on the Patient Health Questionnaire-9 (PHQ-9), altered if ≥5. |  |  |  |  |
|  |  |  |  |  |  |  |  |
|  | Visual, “if distance difficulty, reading difficulty, eye disease, or currently under medical treatment; if the above problems exist, they are considered to be visually impaired; conversely, further use visual acuity chart tests” | Change in visual capacity |  |  |  |  |  |
|  |  |  | Assessment based on clinical interview, need of aids, or presence of diseases. Moreover, visual acuity chart tests (visual test <0.08). |  |  |  |  |
|  | Presence of “communication difficulties caused by hearing, use of hearing aids or related diseases. Instead, use whispering test” | Change in hearing capacity |  |  |  |  |  |
|  |  |  | Assessment based on clinical interview, need of aids, or presence of diseases. Moreover, whispering test (repeated word correctly or not). |  |  |  |  |

**Table 3.** Summary of findings, primary and secondary outcomes, and assessment tools (n = 7 - newest studies first -) *(****part 2/6, continuation***).

**Supplementary material, Table 3S *(part 3/6, continuation****)***:** Summary of findings, primary and secondary outcomes, and measurement tools (n = 7 - newest studies first).

**Table 3.** Summary of findings, primary and secondary outcomes, and assessment tools (n= 7) *(****part 2/5, continuation***).

| **First author, year of publication, journal/registry** | **Primary outcome(s) as quoted by authors** | **Primary outcomes rephrased for ScR purposes**  *Individual items in this column correspond directly to adjacent items in the column on the left and the right. The items related to each domain are grouped. “Change” relates to the change between pre and post-intervention* | **Measurement tools for the primary outcome** | **Secondary outcome(s) as quoted by authors** | **Secondary outcomes rephrased for ScR purposes** *Individual items in this column correspond directly to adjacent items in the column on the left and the right. The items related to each domain are grouped.* “*Change*” *relates to the change between pre and post-intervention* | **Measurement tools for the secondary outcome** | **Comments** *Two columns have been created: .* “*outcomes as quoted by authors*” *and .* “*outcomes rephrased for ScR purposes*”*, for clarity, due to the language gap between the original texts and the rephrased outcomes for review purposes* |
| --- | --- | --- | --- | --- | --- | --- | --- |
| Gimenez Mestre, 2023, Clinicaltrials.gov NCT05744492 | “Change from baseline to 12-weeks in the SPPB''; '' measuring balance, lower extremity strength, and functional capacity in older adults''; same change measured ''from baseline to 12-weeks, from baseline to 6 weeks, and from 12 to 24 weeks” | Change in the locomotor domain, i.e., physical performance | Short Physical Performance Battery (SPPB), /12 | Change in “risk of malnutrition by the MNA-SF (/14)” | Change in nutritional risk status | Mini-Nutritional Assessment Short form (MNA-SF), /14 | None |
|  |  |  |  | Change in “the best attempt of the isometric handgrip strength (kg) of the dominant hand evaluated by a handheld dynamometer'' | Change in muscle strength | Handgrip strength (kg) |  |
|  |  |  |  | Change “from baseline to 12 weeks, from baseline to 6 weeks, and from 12 to 24 weeks in the MoCA, cognitive function (memory, language, attention, executive function, visuospatial and orientation)” | Change in global cognitive function (i.e., memory, language, attention, executive function, visuospatial and orientation) | Montreal Cognitive Assessment (MoCA), /30 |  |
|  |  |  |  | Change in “depressive symptoms by the GDS-15” | Change in the presence and severity of depressive symptoms | Geriatric Depression Scale-15 (GDS-15), /15 |  |
|  |  |  |  | Change in “visual capacity: distance and near vision assessed with WHO simple eye charts” | Change in visual capacity (distance and near vision) | WHO simple eye charts |  |
|  |  |  |  | Change in “hearing capacity measured by HearWHO App consisting of an automated digit-in-noise test” | Change in hearing capacity, understood as an individual's ability to understand speech in noisy environments | HearWHO App, consisting of an automated digit-in-noise test |  |
|  |  |  |  | Change in “frailty status” (objective section) | Change in frailty status | Not described |  |
|  |  |  |  | Change in “health-related quality of life” (objective section) | Change in health-related quality of life | Not described |  |
| Blancafort Alias, 2022, Clinicaltrials.gov NCT05249504 | “Self-perceived health status. Change in self-perceived health status measured by the Euroqol Visual Analogue Scale” | The change in self-perceived health status | Euroqol Visual Analogue Scale, /100 | “Change in functional status according to the Short Physical Performance Battery (SPPB)” | Change in physical performance | Short Physical Performance Battery (SPPB), /12 | The change in cognitive and sensorial aspects were not listed among the outcomes.   Additional information about this study has been obtained from Rojano i Luque et al., 2023 (Available in: https://pubmed.ncbi.nlm.nih.gov/36809987/). |
|  |  |  |  | “Change in the level of physical activity by the Spanish Short Version of the Minnesota Leisure Time Physical Activity Questionnaire (VREM)” | Change in the level of physical activity in the past 14 days | (Spanish Short Version of the) Minnesota Leisure Time Physical Activity Questionnaire (VREM), /≥5000METs-min/14 days |  |
|  |  |  |  | “Change in the ability to perform basic and instrumental daily life activities assessed by the Short-Form Late-Life Function and Disability Instrument (SF-LLFDI)” | Change in the ability to perform basic and instrumental daily life activities and level of autonomy | Short-Form Late-Life Function and Disability Instrument (SF-LLFDI) |  |
|  |  |  |  | “Change in nutritional status, nutritional risks assessed by the MNA-SF” | Change in nutritional risk status | Mini-Nutritional Assessment Short form (MNA-SF), /14 |  |
|  |  |  |  | “Change in the proportion of participants with depressive symptoms assessed by the GDS-5” | Change in the presence and severity of depressive symptoms | Geriatric Depression Scale-5 (GDS-5), /5 |  |
|  |  |  |  | “Change in the degree of loneliness assessed by De Jong Gierveld Loneliness Scale” | Change in the degree of loneliness | De Jong Gierveld Loneliness Scale |  |
|  |  |  |  | “Change in the social support and risk of isolation by the Lubben Social Network Scale-Revised (LSNS-R)” | Change in the social support and risk of isolation | Lubben Social Network Scale - Revised (LSNS-R) |  |
|  |  |  |  | “Change in the health-related quality of life assessed by the EQ-5D-5L” | Change in health-related quality of life | EQ-5D-5L |  |

| **First author, year of publication, journal/registry** | **Primary outcome(s) as quoted by authors** | **Primary outcomes rephrased for ScR purposes**  *Individual items in this column correspond directly to adjacent items in the column on the left and the right. The items related to each domain are grouped. “Change” relates to the change between pre and post-intervention* | **Measurement tools for the primary outcome** | **Secondary outcome(s) as quoted by authors** | **Secondary outcomes rephrased for ScR purposes** *Individual items in this column correspond directly to adjacent items in the column on the left and the right. The items related to each domain are grouped. “Change” relates to the change between pre and post-intervention* | **Measurement tools for the secondary outcome** | **Comments** *Two columns have been created: ''outcomes as quoted by authors'' and ''outcomes rephrased for ScR purposes'', for clarity, due to the language gap between the original texts and the rephrased outcomes for review purposes* |  |
| --- | --- | --- | --- | --- | --- | --- | --- | --- |
| Sánchez-Sánchez, 2022, Age and Ageing | “The composite IC Z-score was constructed, as the sum of the individual z-scores of locomotion, cognition, psychology, and vitality domains divided by 4, to create an IC composite score. GDS score was weighted as -1, because greater scores indicate worse performance in this domain. The sensory domain score was not included in the composite score,...'', 'differences in the evolution of the composite score favouring the intervention group'', ''Individual Z scores were collectively summarized as composite z-score for the primary outcome” | The change in global IC levels (i.e., change in an IC Z-score of the combination of 4 domains: locomotor,vitality, cognitive, and psychological) | An IC Z-score, calculated based on 4 domains' z-scores: locomotor, vitality, cognitive, and psychological   Calculation: the sum of the individual z-scores of locomotor, vitality, cognitive, and psychological domains divided by 4, to create an IC composite score. GDS score was weighted as -1, because greater scores indicate worse performance in this domain. The sensorial domain score was not included in the composite score. Instead, worsening of the IC sensorial domain was characterized as a 1-point increase in the sensorial domain score. | No secondary outcomes were explicitly mentioned in the text, but their results were shown in the results section and the Table 2, after the primary outcome. | Change in the locomotor domain, i.e., the change in the locomotor z-score, expressing the change in physical performance | Locomotor z-score, calculated based on the Short Physical Performance Battery (SPPB) | The individual z-scores for each domain were presented in the results and tables, following the primary outcome. These z-scores have been considered as secondary outcomes for the ScR purposes. |  |
|  |  |  |  |  | Change in physical performance | Short Physical Performance Battery (SPPB), /12 |  |  |
|  |  |  |  |  | Change in the vitality domain, i.e., the change in the vitality z-score, expressing the change in muscle strenght | Vitality z-score, calculated based on the handgrip strenght |  |  |
|  |  |  |  |  |  |  |  |  |
|  |  |  |  |  | Change in muscle strength | Handgrip strength (kg) |  |  |
|  |  |  |  |  | Change in the cognitive domain, i.e., the change in the cognitive z-score, expressing the change in global cognitive function | Cognitive z-score, calculated based on the Montreal Cognitive Assessment (MoCA) |  |  |
|  |  |  |  |  | Change in cognitive function (memory, language, attention, executive function, visuospatial and orientation) | Montreal Cognitive Assessment (MoCA), /30 |  |  |
|  |  |  |  |  | Change in the psychological domain, i.e, the change in the psychological z-score, expressing the change in the presence and severity of depressive symptoms | Psychological calculated based on the Geriatric Depression Scale-15 (GDS-15), /15 |  |  |
|  |  |  |  |  | Change in the presence and severity of depressive symptoms | Geriatric Depression Scale-15 (GDS-15), /15 |  |  |
|  |  |  |  |  | Change in the visual and hearing capacity | A score based on the presence of reduced visual acuity (yes = 1, no = 0), and hypoacusia (yes = 1, no = 0), (range 0–2) |  |  |

**Supplementary material, Table 3S *(part 4/6, continuation****)***:** Summary of findings, primary and secondary outcomes, and measurement tools (n = 7 - newest studies first).

**Supplementary material, Table 3S *(part 5/6, continuation****)***:** Summary of findings, primary and secondary outcomes, and measurement tools (n = 7 - newest studies first).

| **First author, year of publication, journal/registry** | **Primary outcome(s) as quoted by authors** | **Primary outcomes rephrased for ScR purposes**  *Individual items in this column correspond directly to adjacent items in the column on the left and the right. The items related to each domain are grouped. “Change” relates to the change between pre and post-intervention* | **Measurement tools for the primary outcome** | **Secondary outcome(s) as quoted by authors** | **Secondary outcomes rephrased for ScR purposes** *Individual items in this column correspond directly to adjacent items in the column on the left and the right. The items related to each domain are grouped. “Change” relates to the change between pre and post-intervention* | **Measurement tools for the secondary outcome** | **Comments** *Two columns have been created: ''outcomes as quoted by authors'' and ''outcomes rephrased for ScR purposes'', for clarity, due to the language gap between the original texts and the rephrased outcomes for review purposes* |
| --- | --- | --- | --- | --- | --- | --- | --- |
| Giudici, 2020, Maturitas | “The effect of … on levels of intrinsic capacity (IC)'', ''IC Z-score variation'', ''Four IC domains (cognition, locomotion, psychological and vitality) were evaluated to create a unique outcome of IC'', ''IC Z-score decreased among all groups...'', ''x groups found a decrease in IC Z-score after 3 years” | The change in global IC levels (i.e., change in an IC Z-score of the combination of 4 domains: locomotor,vitality, cognitive, and psychological) | An IC Z-score, calculated based on 4 domains' z-scores: locomotor, vitality, cognitive, and psychological  Calculation: A composite IC Z-score was defined as the sum of each domain’s z-scores (standardised with baseline means and standard deviation for each test from the intention-to-treat population) divided by 4, with the psychological domain (GDS) weighted as -1 (since higher scores indicate domain impairment), and all others weighted as 1. | No secondary outcomes were explicitely mentioned | Change in the locomotor domain, i.e., change in the locomotor z-score, expressing change in physical performance | Locomotor z-score, calculated based on the Short Physical Performance Battery (SPPB) | None |
|  |  |  |  |  | Change in physical performance | Short Physical Performance Battery (SPPB), /12 |  |
|  |  |  |  |  | Change in the vitality domain, i.e., change in the vitality z-score, expressing change in muscle strenght | Vitality z-score, calculated based on handgrip strenght |  |
|  |  |  |  |  | Change in muscle strength | Handgrip strength (kg) |  |
|  |  |  |  |  | Change in the cognitive domain, i.e., change in the cognitive z-score, expressing the change in global cognition | Cognitive z-score, calculated based on the 4 z-scores of 4 tests:   - The 10 orientation items of the MMSE - Digit Symbol Substitution test - Free and total recall of the Free and Cued Selective Reminding test - Category Naming test |  |
|  |  |  |  |  | Change in the psychological domain, i.e., change in the psychological z-score, expressing the change in the presence and severity of depressive symptoms | Psychological z-score, calculated based on the Geriatric Depression Scale-15 (GDS-15) |  |
|  |  |  |  |  | Change in the presence and severity of depressive symptoms | Geriatric Depression Scale-15 (GDS-15), /15 |  |

| **First author, year of publication, journal/registry**  **Supplementary material, Table 3S *(part 6/6, end of table****)***:** Summary of findings, primary and secondary outcomes, and measurement tools (n = 7 - newest studies first). | **Primary outcome(s) as quoted by authors** | **Primary outcomes rephrased for ScR purposes**  *Individual items in this column correspond directly to adjacent items in the column on the left and the right. The items related to each domain are grouped. “Change” relates to the change between pre and post-intervention* | **Measurement tools for the primary outcome** | **Secondary outcome(s) as quoted by authors** | **Secondary outcomes rephrased for ScR purposes** *Individual items in this column correspond directly to adjacent items in the column on the left and the right. The items related to each domain are grouped. “Change” relates to the change between pre and post-intervention* | **Measurement tools for the secondary outcome** | **Comments** *Two columns have been created: ''outcomes as quoted by authors'' and ''outcomes rephrased for ScR purposes'', for clarity, due to the language gap between the original texts and the rephrased outcomes for review purposes* |
| --- | --- | --- | --- | --- | --- | --- | --- |
| Huang, 2020, JAMDA | “A general estimating equation was used to analyse mean Z-score change on IC and its domains”, “IC raw score change” (Table 2), “Mean change in composite IC Z-score” (Figure 2), “z-score change in individual domain and combination of all domains”'; “an index reflecting that the higher scores indicate better health”, “the composite IC Z-score, which is the mean of the locomotion z-score, cognition z-score, vitality z-score, and psychological z-score, was the main outcome measure in our analysis” | The change in global IC levels (i.e., change in an IC Z-score of the combination of 4 domains: locomotor,vitality, cognitive, and psychological) | An IC Z-score, calculated based on 4 domains' z-scores: locomotor, vitality, cognitive, and psychological  Calculation: The domain z-score was established by dividing the sum of z-scores from all tests by the number of tests in each domain. To create an index reflecting that the higher scores indicate better health, the positive psychological domain z-score was converted to a negative value | “A general estimating equation was used to analyse mean Z-score change on IC *and its domains*” | Change in the locomotor domain, i.e., change in the locomotor z-score, expressing the change in physical performance | Locomotor z-score, calculated based on 3 tests:   - One-leg stand test (OLS) - 5-meter walking speed (s) - Timed Chair Stand test (s)   Calculation: “as higher scores indicate better health, the z-scores of the completion time for TSTS was converted into a negative value.” | “Cognition z-score was calculated based on sex, age (age 65-74 and ≥75), and educational level stratification.”  “Vitality Z-score derived from handgrip strength considered sex difference.” The calculation and implications of the stratification may be taken into consideration, e.g., a) to confirm that different z-scores were created pending on sex, age, and education, instead of pulling together all the data from each group, b) to discuss the repercussion of the stratifications in the z-scores calculation, c) to discuss how the IC Z-score calculation was affected by the stratification in the domains. |
|  |  |  |  |  | Change in the vitality domain, i.e., change in the vitality z-score, expressing the change in muscle strenght | Vitality z-score, calculated based on muscle strenght  Calculation: “a vitality z-score derived from handgrip strength, and considering sex difference.” |  |
|  |  |  |  |  | Change in the cognitive domain, i.e., change in the cognitive z-score, expressing the change in global cognition, including:   - Immediate memory - Delayed memory - Verbal fluency - Visuospatial memory - Processing speed - Executive function | Cognitive z-score, calculated based on 7 tests:   - Logical Memory I from Wechsler Memory Scale-Revised - Logical Memory II (delayed memory) - Category fluency test and letter fluency test (verbal fluency) - Pentagon copying test (visuospatial memory) - Digit Symbol test (processing speed) - Trail Making Test-part A (processing speed) - Trail Making Test-part B (executive function).”   Calculation: “completion time for Trail Making Test-part A and part B were converted into their opposite values. Cognitive z-score was calculated based on sex, age (age 65-74 and ≥75), and educational level stratification |  |
|  |  |  |  |  | Change in the phycological domain, i.e., change in the psychological z-score, expressing the change in:   - Presence and severity of depressive symptoms - Presence and severity of generalized anxiety symptoms (e.g., excessive worrying, restlessness, and difficulty concentrating) | Phycological z-score, calculated based on 2 tests:   - Geriatric Depression Scale, GDS-15, /15 - Generalized Anxiety Disorder-7 scale (GAD) |  |

Higher values indicate a worst health status in: Fall Efficacy Scale Index, Generalized Anxiety Disorder-7 scale (GAD-7), Geriatric Depression Scale-15 (GDS-15), Patient Health Questionnaire (PHQ-9), Timed Chair Stand Test (TCST), Timed Up and Go Test (TUG), Trail Making Test-part A and Trail Making Test-part part B.
